# Silkworm Cocoon—Derived Carbon Dots for Post-Trauma Hemostasis and Tissue Repair

**DOI:** 10.3390/ph18050603

**Published:** 2025-04-22

**Authors:** Xinru Wu, Miaomiao Yao, Xuan Qiao, Lintao Li, Zhiyun Meng, Shuchen Liu, Yunbo Sun, Hui Gan, Xiaoxia Zhu, Zhuona Wu, Ruolan Gu, Guifang Dou

**Affiliations:** 1 Beijing Institute of Radiation Medicine, Beijing 100850, China; xinru_wu01@126.com (X.W.); yaomiaomiao202109@163.com (M.Y.); 15234444223@163.com (X.Q.); li940408@126.com (L.L.); mengzhiyun@vip.163.com (Z.M.); liusc118@163.com (S.L.); sunyunbo0919@126.com (Y.S.); ganh2003@163.com (H.G.); 13681022512@163.com (X.Z.); wznphd@126.com (Z.W.); 2 School of Pharmaceutical Sciences, Anhui Medical University, Hefei 230032, China

**Keywords:** silkworm cocoon, carbon dots, hemostasis, wound healing, platelets

## Abstract

**Background:** Traumatic hemorrhage management is challenging due to the need to control severe bleeding and support tissue repair. An ideal material would possess both hemostatic and wound-healing properties. **Methods:** Silkworm cocoon-derived carbon dots (SC-CDs) were synthesized via a hydrothermal method. After physical and chemical characterization using techniques such as HR-TEM and XPS, their hemostatic efficacy was assessed in rat liver injury, tail transection, and mouse coagulation disorder models. Moreover, the effects of the SC-CDs on platelet aggregation and activation were evaluated. The potential of the SC-CDs to promote wound healing was investigated through cell scratch assays and a mouse full-thickness skin defect model. **Results:** The SC-CDs showed a high quantum yield (12.9% ± 0.42%), with low hemolytic activity and cytotoxicity. In the hemostasis models, the SC-CDs significantly reduced the bleeding time and volume. In the rat liver injury model, the bleeding time was shortened from 152.67 ± 4.16 s (Control) to 55.33 ± 9.50 s (*p* < 0.05). In the rat tail transection model, the bleeding volume was reduced from 1.71 ± 0.16 g (Control) to 0.4 ± 0.11 g (*p* < 0.05). In the mouse coagulation disorder model, an 8 mg/kg dose reduced the bleeding volume to 11.80% ± 0.39% of that of the Control (*p* < 0.05). Mechanistic studies suggested enhanced platelet activation and aggregation. In the wound healing experiments, the SC-CDs reduced the wound area (88.53 ± 11.78 mm^2^ (Control) vs. 70.07 ± 6.71 mm^2^ (SC-CDs), *p* < 0.05) and promoted fibroblast migration (24 h scratch width: 372.34 ± 9.06 μm (Control) vs. 259.49 ± 36.75 μm (SC-CDs), *p* < 0.05). **Conclusions:** SC-CDs show promise for hemorrhage management and tissue regeneration, with potential applications in cases of internal bleeding or coagulation disorders.

## 1. Introduction

Globally, traumatic hemorrhage causes approximately 2.5 million deaths annually, with complications such as hemorrhagic shock, coagulation dysfunction, and sepsis posing significant risks [1]. Currently, several different types of topical hemostatic materials are available for the treatment of external bleeding, including zeolite [2], kaolin [3], and chitosan [4]. Injectable hemostatic drugs primarily include thrombin [5], tranexamic acid [6], and vasoconstrictors [7], which directly or indirectly affect the coagulation system and complement functional composite hemostatic materials. However, effective intravenous interventions for internal/organ bleeding remain limited. Moreover, massive blood loss often depletes coagulation factors, necessitating high-efficacy hemostatic agents. In addition to hemostatic activity, the ability to manage late-stage tissue healing is necessary for the control of traumatic bleeding, as wound healing can be further complicated by secondary complications, such as microbial infection [8]. Therefore, ideal materials should facilitate rapid hemostasis and promote wound healing to prevent infection.

Silkworm cocoons have long been used in traditional Chinese medicine because of their hemostatic properties. Ancient medical texts state that silkworm cocoons were commonly used as hemostatic agents after undergoing various processing steps, such as boiling at high temperatures or incineration to ash, followed by either oral ingestion or external application [9]. Silkworm cocoons are predominantly composed of fibroin and sericin, which account for approximately 75–83% and 17–25% of their weight, respectively [10]. In addition, silkworm cocoons contain trace amounts of nonsericin components, including flavonoids (such as quercetin and kaempferol) [11], alkaloids [12], coumarin derivatives [13], and other small molecules.

Recent studies have examined the hemostatic and wound-healing properties of fibroin and sericin proteins [14,15]. A combined extract of sericin and fibroin has demonstrated significant procoagulant activity in vitro [16]. Because of its high biocompatibility and mechanical strength, sericin can enhance the wound-healing process [17]. Researchers have developed a fibroin-based injectable sealant to facilitate rapid hemostasis and promote wound healing [18], whereas others have created a sericin/fibroin protein aerogel that aids in hemostasis through physical and biological mechanisms [19]. Protein components derived from silkworm cocoons exhibit considerable potential for application in hemostasis and wound healing.

When exposed to denaturing factors such as heat [20] and organic solvents [21], these proteins undergo disruption of their internal secondary bonds, which leads to alterations in their three-dimensional structures and consequently affects their functional capabilities. High-temperature treatment can degrade proteins such as sericin [22]. Specific molecular interactions may provide some degree of structural and functional protection to these proteins [23]. However, during carbonization, the absence of protective agents within silkworm cocoons typically results in unavoidable protein denaturation. Therefore, it is possible that the substances within silkworm cocoons contributing to their hemostatic effects following high-temperature treatment do not rely solely on functional components, such as fibroin and sericin.

The synthesis methods for carbon dots closely resemble the processing techniques used in traditional Chinese medicine [24], which primarily include hydrothermal, pyrolysis, and microwave approaches [25]. Carbon dots produced through the hydrothermal method exhibit improved water solubility and biocompatibility [26,27]. Recent studies have indicated that traditional Chinese medicines subjected to high-temperature carbonization can yield carbon quantum dots with diameters of <10 nm, which are referred to as traditional Chinese medicine-derived carbon dots [28,29,30]. These structures consist of two components: sp^2^/sp^3^ amorphous carbon atom cores and shells enriched with O/N/S functional groups or polymer chains [31]. They show excellent biocompatibility, water solubility, and low toxicity [32] and are readily functionalized. As relatively safe nanomedicines with pharmacological activity, many carbon dots inherit the biological functions of their precursors or exhibit novel pharmacological properties [27]. Table 1 lists the biological activities of carbon dots derived from partial biomass or natural macromolecule sources. Based on the potential biological applications of silkworm cocoons and their components, we hypothesized that SC-CDs can contribute to hemostasis and wound-healing promotion, with carbon dots playing an important role in mediating pharmacological activities after incinerating silkworm cocoons into ash.

Carbon dots were successfully synthesized from silk cocoons using a hydrothermal method. High quantum yield SC-CDs were obtained through dialysis and high-speed centrifugation. The physical and chemical properties of the SC-CDs were characterized using various techniques, including assessments of morphology, structure, chemical composition, and optical properties. To determine the pharmacological activities of the SC-CDs, we assessed their hemostatic and wound-healing properties from multiple perspectives in vivo and in vitro. The hemostatic effects of the SC-CDs on internal bleeding, traumatic bleeding, and bleeding resulting from coagulation disorders were evaluated using a rat liver injury model, a tail transection model, and a mouse snake venom-induced bleeding model. In addition, four coagulation parameters were measured, and the effect of the SC-CDs on platelets was examined in vitro to preliminarily elucidate the underlying coagulation mechanisms. Furthermore, cell scratch assays and mouse full-thickness skin defect models were used to assess the wound-healing effects of the SC-CDs and explore the mechanisms involved. The results provide insights into the potential medical applications of SC-CDs, particularly in hemostasis and wound healing.

## 2. Results

### 2.1. Synthesis and Characterization of SC-CDs

**Figure 1:**
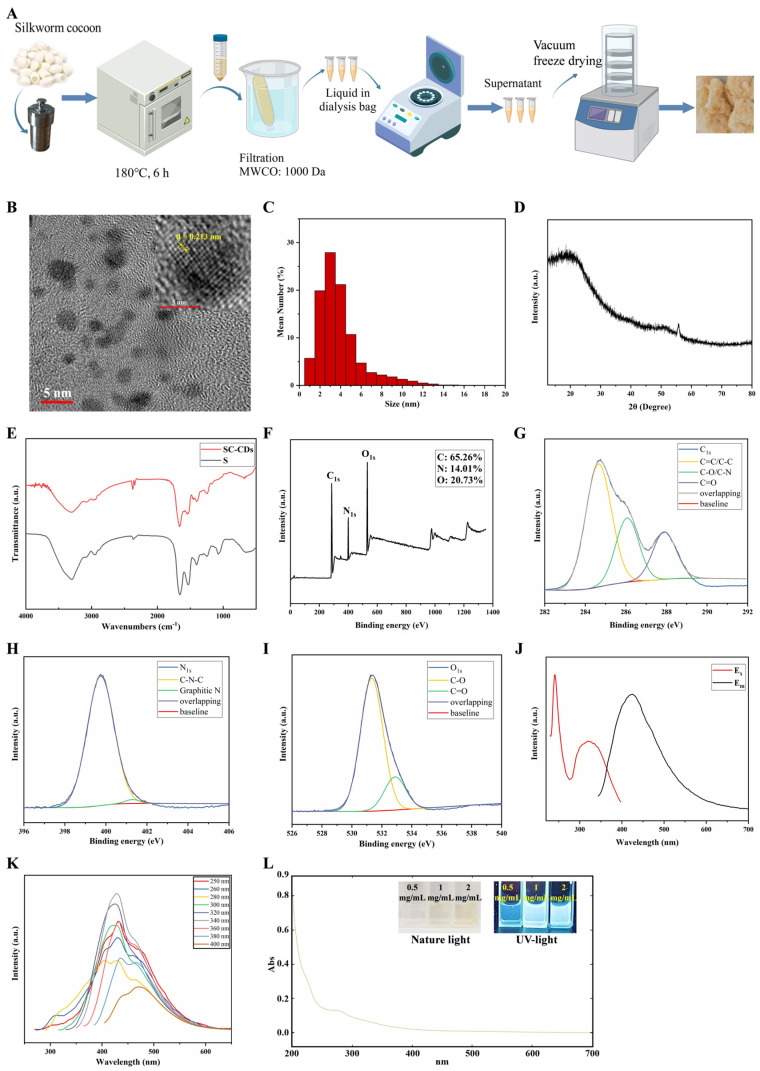
Synthesis and characterization of SC-CDs. Preparation of SC-CDs (**A**). HR-TEM (**B**) and particle size distribution (**C**) of SC-CDs. XRD (**D**) and FTIR (**E**) characteristics of SC-CDs. XPS spectrum of SC-CDs (**F**–**I**). Full spectrum (**F**). Fine spectra of C1s (**G**), N1s (**H**), and O1s (**I**). Fluorescence (**J**,**K**) and UV (**L**) absorption spectra.

The synthesis temperature and duration for SC-CDs were optimized using a hydrothermal method based on the quantum yield and hemostatic activity of the resulting products (Figure S1A–D). The SC-CDs with the highest quantum yield and enhanced coagulation activity were successfully synthesized under conditions of 180 °C for 6 h. Consequently, the optimal synthesis parameters for SC-CDs were established. After synthesizing the light-yellow SC-CDs solution using the hydrothermal method (Figure 1A), it was subjected to vacuum freeze-drying to obtain SC-CDs powder. Initially, both the physical and chemical properties were characterized. High-resolution transmission electron microscopy (HR-TEM) was used to examine the surface morphology of the carbon dots (Figure 1B), which revealed a spherical structure with a lattice spacing of 0.213 nm. The particle size distribution analysis (Figure 1C) revealed a predominant size range of 2–3 nm and uniform dispersion, suggesting enhanced membrane permeability of the SC-CDs because of their nanoscale dimensions [25,45]. Further structural characterization of the SC-CDs was performed using X-ray diffraction (XRD) analysis. The broad peak at 20° in Figure 1D corresponds to the amorphous structure of carbon dots and is attributed to the (002) crystal plane of graphene [46], which is consistent with the HR-TEM results. In the Raman spectrum (Figure S2A), the peaks observed at 1351 and 1564 cm^−1^ corresponded to the disordered D peak and ordered G peak [47], respectively, indicating the coexistence of an amorphous structure and an ordered carbon core in the SC-CDs. The intensity ratio between the D and G peaks was 0.97. These findings suggest that the SC-CDs comprise a composite of graphitic carbon and amorphous carbon and exhibit a discernible degree of crystallinity.

The surface functional groups of the SC-CDs powder obtained through vacuum freeze-drying were analyzed using Fourier-transform infrared spectroscopy (FTIR) (Figure 1E). Compared with the silkworm cocoon decoction extract, the SC-CDs exhibited enhanced retention of sulfur-containing functional groups, such as the C–O stretching vibration at 1245 cm^−1^ and the C=O stretching vibration at 1659 cm^−1^. However, the peak representing the C–O–C bond stretching vibration at 1075 cm^−1^ in the S spectrum was absent in the SC-CDs. Notably, new peaks emerged in the SC-CDs, including one at 1397 cm^−1^, indicating C–H bending vibrations, and another at 3300 cm^−1^, which was attributed to the stretching vibrations of O–H or N–H bonds, thereby demonstrating the excellent water solubility of the SC-CDs [48]. In addition, X-ray photoelectron spectroscopy (XPS) was used to analyze the elemental surface composition and functional groups present on the surface of the SC-CDs (Figure 1F–I). The percentage distribution of the elements C, N, and O was approximately 65.26%, 14.01%, and 20.73%, respectively (Figure 1F), whereas the high-resolution C_1s_ spectrum (Figure 1G) exhibited distinct peaks corresponding to different carbon functionalities: 284.66 eV for C=C/C–C; 286.08 eV for C–O/C–N; and 287.91 eV for C=O [49]. The N_1s_ spectrum (Figure 1H) exhibited a peak at 399.75 eV, corresponding to C–N–C bonding, and the minor presence of graphitic nitrogen was also observed with a peak at 401.31 eV. Figure 1I shows the high-resolution O_1s_ spectrum, revealing absorption peaks at 531.33 and 532.9 eV, which were attributed to C–O and C=O absorption [50], respectively. These XPS results corroborated the FTIR data, confirming the surface functional groups and elemental composition of the carbon dots, which serve as a structural foundation for the SC-CDs’ activity. Moreover, zeta potential measurements revealed that the SC-CDs contain a negative charge with a potential of −7.46 mV (Figure S2B), which can promote coagulation by activating the intrinsic coagulation pathway [51].

The fluorescence spectrum of the SC-CDs is illustrated in Figure 1J, which shows an emission wavelength of 430 nm upon excitation at 340 nm. Variations in the magnitude of the fluorescence intensity and emitted wavelength were observed across different emission wavelengths (Figure 1K), indicating an excitation-dependent emission behavior [33]. The ultraviolet–visible spectrum illustrated in Figure 1L shows the broad absorption profile of the SC-CDs with respect to their optical properties. The spectrum showed a weak absorption peak at 270 nm, which was attributed to the π–π* electronic transition of the aromatic sp^2^ structure present in the SC-CDs [52]. This finding was consistent with that of previous studies on carbon dots [41,53]. The quantum yield (QY) of carbon dots refers to the ratio of the number of emitted photons to the number of absorbed photons per unit of time under excitation at a specific wavelength. A positive correlation exists between QY and purity [54]. Using the hydrothermal method, we obtained SC-CDs with a QY of 12.9%. Our hydrothermally synthesized product exhibited a higher QY than the carbon dots synthesized by Wang et al. from silkworm cocoons via pyrolysis [39].

### 2.2. In Vitro Biocompatibility of SC-CDs

#### 2.2.1. Hemocompatibility of SC-CDs

**Figure 2:**
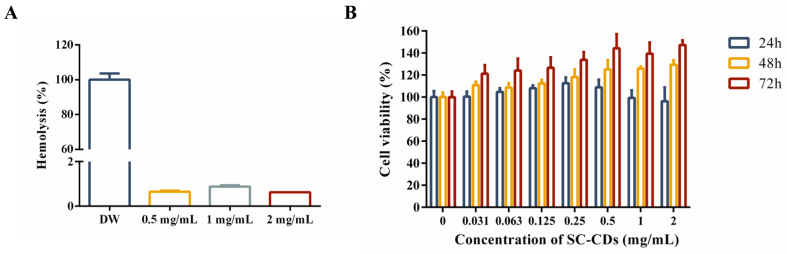
In vitro biocompatibility and cytotoxicity assessment of SC-CDs. Hemolysis (**A**) (*n* = 3) and cytotoxicity of SC-CDs on L929 cells (**B**) (*n* = 6).

To evaluate the hemocompatibility of the SC-CDs and their effect on blood homeostasis, a hemolysis test was performed using red blood cells. The experiment used three SC-CDs concentrations (0.5, 1, and 2 mg/mL) to assess the blood compatibility of the carbon dots (Figure 2A,B). Compared with the positive control group, the SC-CDs group exhibited a minimal hemolysis rate of approximately 0.65% ± 0.05%, indicating a negligible impact on blood function. This favorable hemocompatibility highlights the potential clinical applicability of SC-CDs [55].

#### 2.2.2. Cytotoxicity of SC-CDs in L929 Cells

The assessment of cytotoxicity has significant implications for the safety of drugs administered in vivo. The cytotoxicity of the SC-CDs was evaluated using L929 cells (Figure 2B). The experimental concentrations of the SC-CDs ranged from 0.031 to 2 mg/mL. Under these conditions (0.031–2 mg/mL), the SC-CDs were nontoxic to L929 cells, as evidenced by the consistently high survival rates (>95%), which is consistent with the well-established biocompatibility of carbon dots.

### 2.3. In Vitro Bioactivity of SC-CDs

#### 2.3.1. In Vitro Hemostatic Activity

**Figure 3:**
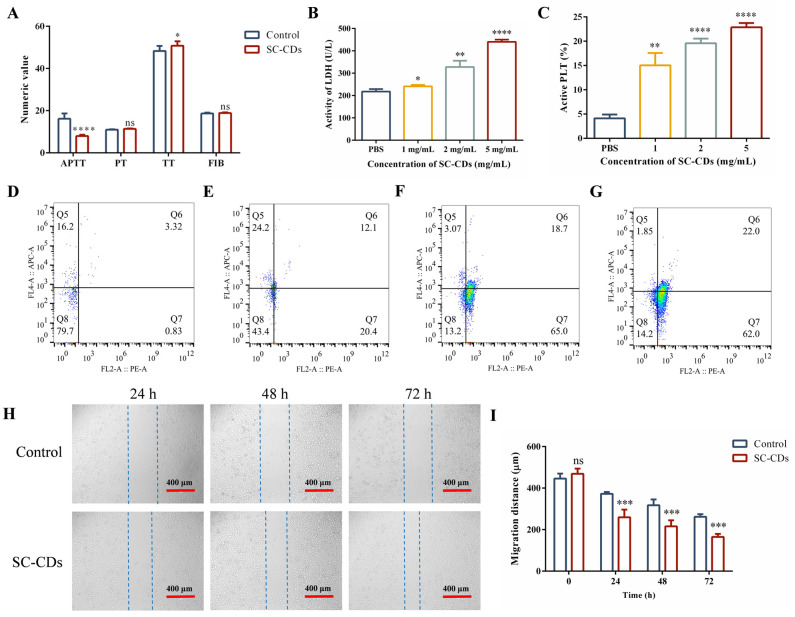
In vitro activity of silkworm cocoon-derived carbon dots (SC-CDs). Coagulation activity (coagulation parameters (**A**), platelet aggregation (**B**), and platelet activation (**C**–**G**)) (n = 3). L929 cell migration (**H**) and migration rate (**I**) (n = 6). * *p* < 0.05, ** *p* < 0.01, *** *p* < 0.001, **** *p* < 0.0001, ns: *p* > 0.05.

Figure S1B,D demonstrate the in vitro hemostatic efficacy of the SC-CDs. A preliminary evaluation of the procoagulant mechanism of the SC-CDs was conducted (Figure 3A). The coagulation parameters, including activated partial thromboplastin time (APTT), prothrombin time (PT), thrombin time (TT), and fibrinogen (FIB), representing different coagulation pathways are shown. The APTT primarily reflects the intrinsic coagulation pathway involving clotting factors XII and XI. The TT primarily indicates fibrinogen functionality, whereas the PT reflects the extrinsic coagulation pathway. Compared with the Control group, the SC-CDs group primarily affected the plasma by shortening the APTT and slightly increasing the TT, indicating coagulation activation through the intrinsic pathway with a slight influence from the negative charge on the surface of the SC-CDs. Overall, these results demonstrate the rapid blood clotting achieved through the influence of the SC-CDs on the APTT [56]. Hemostasis and thrombosis rely heavily on platelet aggregation and activation [57]. The quantitative analysis of adherent platelets using a lactate dehydrogenase (LDH) assay (Figure 3B) revealed significant enhancement in the proaggregation effect of the SC-CDs at all concentrations compared with the Control group. This indicates that the SC-CDs promote platelet aggregation during hemostasis. Flow cytometry enabled the quantitative assessment of the platelet activation induced by the SC-CDs. As shown in Figure 3C–G, different concentrations of the SC-CDs activated platelets in a dose-dependent manner, with 1, 2, and 5 mg/mL of the SC-CDs activating 15.03% ± 2.55%, 19.6% ± 0.95%, and 22.87% ± 0.90% of total platelets, respectively. However, the Control group exhibited a platelet activation rate of only 4.14% ± 0.78%. Compared with the Control group, the SC-CDs significantly enhanced the aggregation and adhesion of platelets, thereby playing a crucial role in initiating thrombus contraction and fibrin formation [10].

#### 2.3.2. Cell Wound Scratch Assay

The scratch assay is widely used for assessing the in vitro activity of test substances by measuring cell migration, which is a surrogate for their potential to promote wound healing [58]. As shown in Figure 3H,I, treatment of L929 cells with 500 μg/mL of the SC-CDs significantly reduced the scratch width compared with that of the Control group. After 24 h, the Control group exhibited a scratch width of 372.34 ± 9.06 μm, whereas the SC-CD group showed a width of 259.49 ± 36.75 μm. Following a 72 h treatment, the width in the Control group decreased to 261.77 ± 12.26 μm, whereas that in the treated group reached 164.89 ± 14.45 μm. Notably, treatment with 500 μg/mL of the SC-CDs significantly enhanced L929 cell migration. This effect became more pronounced as the treatment duration extended to 72 h, which indicates that they may have a positive effect on wound healing.

### 2.4. In Vivo Hemostatic Activity of SC-CDs

#### 2.4.1. Hemostatic Effect of SC-CDs on Acute Hemorrhage in Rats

**Figure 4:**
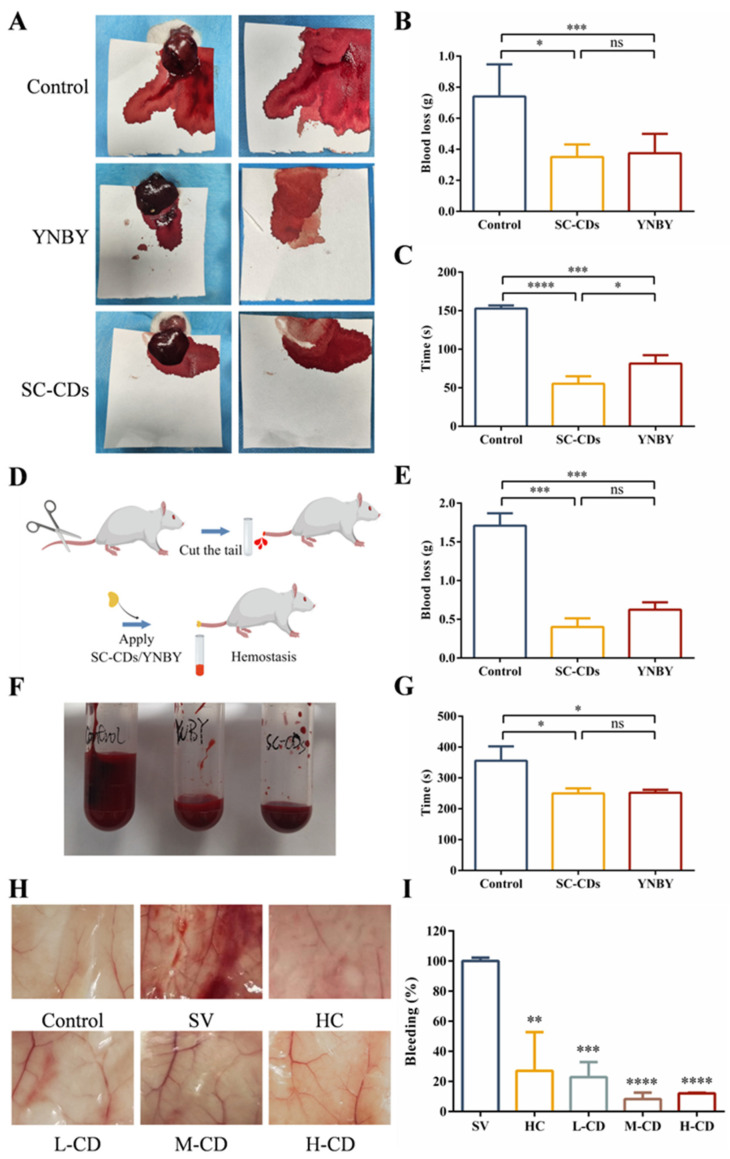
In vivo hemostatic activity of silkworm cocoon-derived carbon dots (SC-CDs). Images of liver injury, bleeding, and hemostasis in the Control, SC-CD, and YNBY groups of rats (**A**), blood loss (**B**), and hemostasis time (**C**). Flowchart of the tail amputation bleeding model (**D**), images of tail bleeding in different treatment groups (**E**), blood loss (**F**), and hemostasis time (**G**). Schematic diagram of peritoneal bleeding in different treatment groups in the snake venom-induced bleeding model (**H**) and the relative peritoneal blood loss (**I**). (n = 3) * *p* < 0.05, ** *p* < 0.01, *** *p* < 0.001, **** *p* < 0.0001, ns: *p* > 0.05.

This experiment established a rat liver injury model to assess the hemostatic effect of the SC-CDs on acute hemorrhage. To enhance the applicability of the SC-CDs in acute bleeding scenarios, the hemostatic efficacy of powdered SC-CDs was assessed. Figure 4A–C depicts the results of the hemostasis experiment in a rat liver injury model. After exposing the left lobe of the rat liver and placing it flat on a filter paper, a 1 cm long and 0.3 cm deep wound was created using a needle. Following 5 s of free bleeding, 30 mg of the SC-CDs or YNBY powder was immediately sprinkled onto the wound site while applying light pressure with a weight to prevent powder loss. The blood coagulation time was recorded. Subsequently, the filter paper was weighed to measure the amount of blood lost (Figure 4B,C). The bleeding time of the Control group was 153.67 ± 4.16 s, and the amount of blood lost was 0.74 ± 0.21 g. The SC-CDs group exhibited a significantly reduced bleeding time (55.33 ± 9.50 s) compared with the YNBY group (81.33 ± 10.97 s). In addition, the amount of bleeding was 0.35 ± 0.08 g in the SC-CDs group and 0.41 ± 0.16 g in the YNBY group. These findings indicate that SC-CDs can alleviate the physical damage associated with traumatic bleeding by reducing the duration of hemorrhaging and the total amount of blood lost.

#### 2.4.2. Coagulation Capacity in a Rat Model of Tail Amputation Hemorrhage

The results are presented in Figure 4D–F. The SC-CDs were assessed in a powdered state to simulate rapid hemostasis in a compressible bleeding scenario. Following mid-tail transection of the rat’s tail, 20 mg of the SC-CDs or YNBY powder was topically applied to the wound site. The bleeding volume was quantified using preweighed 5 mL Eppendorf tubes, and the time to blood flow cessation and the amount of bleeding were recorded (Figure 4E,F). The Control group showed an extended bleeding time (355.67 ± 46.70 s) and increased blood loss (1.71 ± 0.16 g). In contrast, the SC-CDs group exhibited significantly reduced bleeding times (249.67 ± 16.56 s) and blood loss (0.40 ± 0.11 g). Moreover, the SC-CDs group exhibited consistent hemostatic effects that were comparable to those observed in the YNBY group (bleeding time: 251.67 ± 9.81 s; blood loss: 0.62 ± 0.10 g). These results indicate that SC-CDs can promote rapid hemostasis and decrease bleeding during tail-cutting procedures in rats.

#### 2.4.3. Hemostasis of SC-CDs in Snake Venom-Induced Bleeding

The venom produced by *Deinagkistrodon acutus* can induce coagulation disorders, resulting in symptoms such as bleeding [16]. Antiplatelet peptides present in venom can inhibit platelet aggregation and reduce platelet counts, leading to thrombocytopenia and abnormal coagulation pathways [59]. To determine the hemostatic effect of the SC-CDs, bleeding was induced in mice through the intraperitoneal injection of snake venom. This experiment was performed to assess the hemostatic efficacy of an intraperitoneal injection of the SC-CD solution. Figure 4G shows the significant inhibitory effect of the SC-CDs on the peritoneal bleeding induced by snake venom. Figure 4H,I illustrate the occurrence of abdominal hemorrhage in mice. The Control group, which did not receive snake venom injections, exhibited no signs of abdominal hemorrhage. Compared with the snake venom (SV) group, all doses of the SC-CDs significantly reduced the extent of abdominal hemorrhage. Specifically, the amount of hemorrhage observed in the L-CD, M-CD, and H-CD treatment groups was 17.05% ± 10%, 5.93% ± 4.24%, and 11.80% ± 0.39% of that of the SV group, respectively.

### 2.5. Wound-Healing Effect of SC-CDs

#### 2.5.1. Change in Wound Area in a Mouse Model

**Figure 5:**
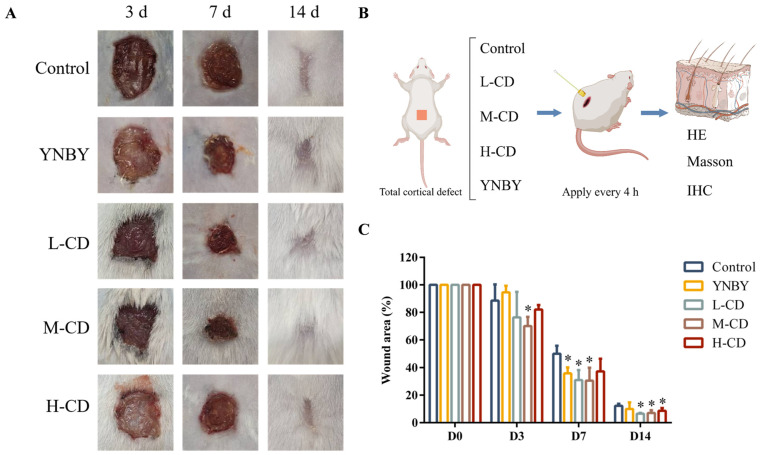
Wound-healing activity of silkworm cocoon-derived carbon dots (SC-CDs). Images of healing wounds from different treatment groups on days 3, 7, and 14 (**A**); schematic diagram of process (**B**); and wound-healing rate (**C**) (n = 3). * *p* < 0.05.

The wound-healing capability of the SC-CDs was evaluated in a mouse model of a full-thickness skin defect (Figure 5A). The wound-healing progress was monitored and tissue samples were collected for histomorphometric analysis on days 0, 3, 7, and 14. As shown in Figure 5B,C, various concentrations of the carbon dots exhibited a favorable effect on wound healing. Notably, the M-CD group showed the most significant wound-healing response, with the highest value (70.07% ± 6.71%) on day 3. Interestingly, even after cessation of drug administration, the wound healing in each SC-CDs concentration group continued to surpass that observed in the Control and YNBY groups. This may be attributed to the antioxidant properties of the SC-CDs, which are capable of eliminating excessive reactive oxygen species (ROS) at the wound site (Figure S3A,B) [60].

#### 2.5.2. HE Staining and Histopathological Scoring of the Wound Surface

**Figure 6:**
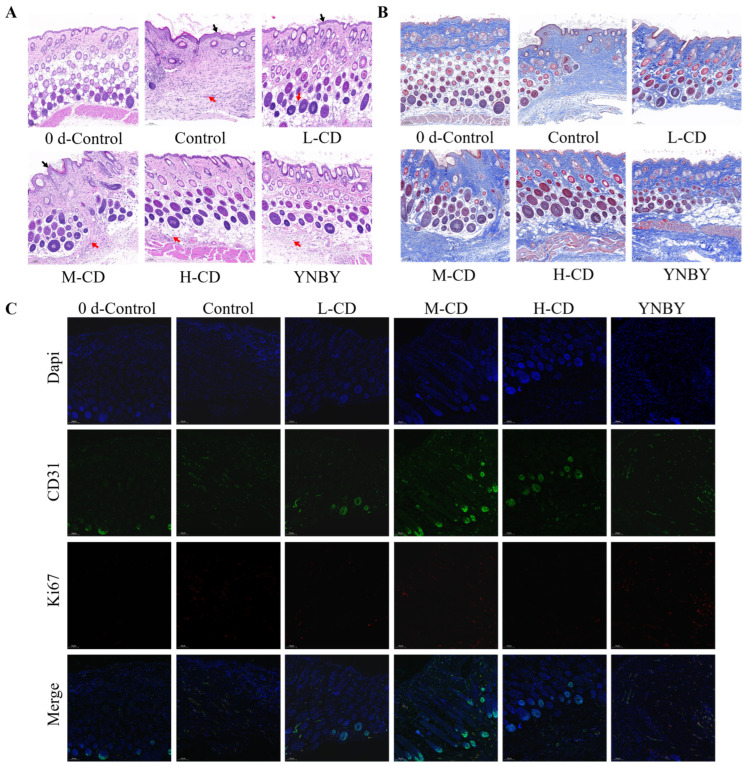
Wound tissue in the Control, SC-CD, and YNBY treatment groups was visualized using HE staining (**A**) and Masson staining (**B**), and immunohistochemical analysis was used to detect Ki67 and CD31 (**C**), (black arrows: epidermis, red arrows: vessels) (100×).

Pathological and histological assessments of the wound surface were performed, and the results of the HE staining are shown in Figure 6A. Compared with normal skin on day 0, the Control group on day 14 exhibited an aberrant basic skin structure characterized by significant epidermal thickening (indicated by black arrows) and a loss of local hair follicles and appendages. The L-CD, M-CD, and H-CD groups exhibited a normal basic skin structure with some epidermal thickening, enhanced neovascularization (indicated by red arrows), and dense collagen networks and hair follicles. During the initiation of wound healing, collagen rapidly accumulates within the skin tissue, contributes to granulation tissue formation, and participates in the tissue-remodeling phase. Collagen not only provides structural support for wound healing but also significantly affects the cells it is in contact with [11].

Masson staining was used to differentiate the collagen and muscle fibers within the tissue. The results illustrated in Figure 6B demonstrate a significant enhancement in collagen fiber deposition across all the experimental groups compared with that on day 0. Specifically, the SC-CDs group exhibited a markedly higher level of collagen deposition than the Control group.

CD31, also known as platelet endothelial cell adhesion molecule 1, acts as a blood vessel and endothelial cell marker [12]. Ki67 serves as a cell proliferation marker that is used to identify actively dividing cells [13]. Figure 6C shows the outcomes of immunohistochemical staining for CD31 and Ki67. Although no significant differences in Ki67 levels were observed between the groups, treatment with the SC-CDs resulted in a marked increase in CD31 expression at the wound site, suggesting enhanced formation of new blood vessels.

## 3. Discussion

The SC-CDs synthesized through the hydrothermal method in this study exhibit a uniform size ranging from 2 to 3 nm and possess an amorphous carbon core enriched with oxygen and nitrogen functional groups (–OH, –COOH, and C–N–C), as confirmed by the HR-TEM, XPS, and FTIR analyses. This structural characteristic is consistent with that of carbon dots derived from natural precursors such as *Platycladus orientalis* [33] and *Ligusticum wallichii* [34]. The SC-CDs demonstrated a higher quantum yield of 12.9%, significantly outperforming pyrolytically synthesized silkworm cocoon-derived carbon dots (6.32%) [39], thereby indicating their superior photoelectric performance. Furthermore, the surface charge of the SC-CDs was measured to be −7.46 mV, which is a critical attribute for activating the endogenous coagulation pathway within biological systems [51]. These physicochemical properties not only enhance water solubility and biocompatibility, but also establish a dual functional basis [61] for SC-CDs in hemostasis and wound healing.

The in vitro coagulation experiments demonstrated that the SC-CDs significantly shortened the APTT, indicating a preferential activation of the intrinsic coagulation pathway [62]. This effect was attributed to their negatively charged surface, which can mimic the physiological role of collagen in recruiting factor XII upon exposure to anionic phospholipids [57]. Further platelet activation experiments revealed that the SC-CDs enhanced both platelet aggregation (PBS: 217.89 ± 11.23 U/L; SC-CDs: 440.86 ± 9.84 U/L) and activation (PBS: 4.14 ± 0.78%; 5 mg/mL SC-CDs: 22.87 ± 0.90%). Similar platelet aggregation-promoting activity was observed in carbon dots derived from *Platycladus orientalis*, which activated platelets via the Src/Syk signaling pathway [33].

The results of the in vivo experiments demonstrated that the SC-CDs exhibited comparable efficacy to the clinically approved hemostatic powder Yunnan Baiyao [63] (YNBY) in terms of reducing blood loss (liver injury: 0.74 ± 0.21 g vs. 0.90 ± 0.08 g; tail transection: 1.71 ± 0.16 g vs. 0.40 ± 0.11 g). In the rat liver injury bleeding model, the SC-CDs achieved a faster hemostasis speed than YNBY (152.67 ± 4.16 s vs. 55.33 ± 9.50 s). This enhanced performance may be attributed to the nanoscale size and excellent water solubility of the SC-CDs, which facilitate their penetration into wound crevices and blood [64], whereas YNBY particles are micrometer-sized and exhibit high hydrophobicity [2]. In comparison with hemocoagulase, a clinical treatment for snake venom, the SC-CDs demonstrated a similar hemostatic effect while potentially mitigating the risks associated with thrombin allergy and hypofibrinogenemia [65].

In the full-thickness skin defect model, the SC-CDs significantly accelerated the wound-closure process in mice, achieving healing effects comparable to those of YNBY during the recovery period. Carbon dots facilitate wound closure by alleviating oxidative stress through ROS scavenging, inhibiting bacterial proliferation within wounds, and promoting fibroblast migration [60], which is consistent with the findings reported by He et al. [66]. The immunohistochemical analysis revealed an increased expression of CD31 at the wound site in the SC-CDs treatment group, indicating that the SC-CDs promoted angiogenesis [12]. This characteristic is notably absent in traditional chitosan-based dressings [67].

In terms of biosafety, the SC-CDs exhibit excellent blood compatibility, with a hemolysis rate of 0.65% ± 0.05% and negligible cytotoxicity manifested, as indicated by the >95% viability in L929 cells at a 2 mg/mL concentration, consistent with the established safety profile associated with carbon dots [32]. Their small size and hydrophilic surface groups suggest that renal clearance serves as the primary excretion pathway, thereby minimizing the risk of long-term organ accumulation [45]. Zhang et al. investigated the metabolic distribution of carbon dots derived from *Punica granatum* L. in mice and found that these particles could be completely excreted within 10 h, indicating favorable biocompatibility [68]. Future studies should prioritize assessing the biodistribution and chronic toxicity of SC-CDs, particularly considering potential accumulation in organs and tissues following repeated administration [27].

Compared to traditional formulations, SC-CDs are particularly well-suited for a range of application environments, including intraperitoneal injection and localized use. The efficacy of SC-CDs for injection purposes was demonstrated in an intraperitoneal injection model utilizing snake venom, which underscored their advantages over vasoconstrictor-based injectables that carry risks of tissue ischemia [7]. While in situ gels provide sustained drug release [18], the liquid-to-powder versatility of SC-CDs makes them applicable for both local and intraperitoneal administration.

This study experimentally validated the biological activities of SC-CDs in promoting hemostasis and wound healing. Future research should focus on elucidating the detailed molecular mechanisms underlying the hemostasis and wound healing mediated by SC-CDs; investigating their pharmacokinetics and longitudinal biosafety profiles in vivo; as well as optimizing their formulation for clinical translation.

## 4. Materials and Methods

### 4.1. Materials

Silkworm cocoons were collected in Xi’an, Shanxi Province, China. The APTT test kit (catalog number: R01101; batch number: 20231103M), PT test kit (catalog number: R07901, batch number: 20231103M), TT test kit (catalog number: R08001; batch number: 20231024M), and FIB test kit (catalog number: R08101; batch number: 20231211M) were procured from Shenzhen LDT Life Science Co., Ltd. (Shenzhen, Guangdong, China). Dulbecco’s Modified Eagle Medium (DMEM) (catalog number: C11995500BT; batch number: 6123074) and fetal bovine serum (catalog number: FSD500; batch number: 2206993CP) were sourced from Thermo Fisher Scientific (China) Co., Ltd. (Shanghai, China). The APC anti-mouse/rat CD61 antibody (catalog number: 108303; batch number: B388477) and PE anti-mouse/rat CD62P antibody (catalog number: 104307; batch number: B370603) were acquired from Biolegend (San Diego, CA, USA). The snake venom was provided by the Snakebite Research Center of the Anhui Academy of Chinese Medicine Sciences (Hefei, Anhui, China). The Agkistrodon acutus venom hemocoagulase injection was obtained from Jinzhou Aohong Pharmaceutical Co., Ltd. (Jinzhou, Liaoning, China). All other chemical reagents were analytical grade.

### 4.2. Cells and Animals

L929 cells (catalog number: SCSP-5039; ATCC No.: CCL-1™) were purchased from the Chinese Academy of Sciences Stem Cell Bank. Male Sprague–Dawley rats weighing 200 g and male BALB/c mice weighing 20 g were procured from the Beijing Keyu Animal Breeding Center.

### 4.3. Preparation of SC-CDs

Silkworm cocoons (0.8 g) were mixed with 40 mL of water in a reaction kettle and incubated at 180 °C for 6 h to obtain a solution containing carbon dots. The solution was then filtered through a membrane with a pore size of 0.45 μm, followed by dialysis for 72 h using a dialysis bag (catalog number: HF132640; Beijing Ruida Henghui Technology Development Co., Ltd., Beijing, China) with a molecular weight cutoff of 1000 Da; the water was changed every 12 h. Subsequently, the dialyzed carbon dot solution was centrifuged at 11,000 r/min for 30 min to obtain purified carbon dots. This solution was then pre-frozen at –20 °C for 12 h. In contrast to conventional freeze-drying processes conducted under normal or slightly negative pressure, this experiment utilized a vacuum freeze dryer (LGJ-10, Beijing Songyuan Huaxing Technology Development Co., Ltd., Beijing, China) to create a vacuum environment of 0.001 kPa for drying the SC-CDs solution. This method enabled the preparation of SC-CDs powder with preserved structural integrity while maintaining the native bioactivity of the SC-CDs.

### 4.4. Characterization of SC-CDs

The spectroscopic properties of the SC-CDs were characterized using a UV spectrophotometer (UV-2600; Shimadzu, Kyoto, Japan) and a fluorescence spectrophotometer (FLS1000; Guangzhou Beta Technology Co., Ltd., Guangzhou, Guangdong, China). The QY of the SC-CDs was determined at an excitation wavelength of 241 nm and an emission wavelength of 422 nm. Fourier-transform infrared spectroscopy (FTIR-1500; Zhongshuo Ke (Tianjin) Technology Development Co., Ltd., Tianjin, China) was used to analyze the unique carbon dot composition within the wavelength range of 40–4000 cm^−1^. The surface elemental composition and associated chemical groups of the SC-CDs were analyzed via XPS (ESCALAB 250Xi; Thermo Fisher Scientific, Waltham, MA, USA). The particle size distribution was determined using a Malvern laser particle size analyzer (Zetasizer Nano ZS; Malvern Instruments Ltd., Malvern, Worcestershire, UK). To assess the structural regularity of the SC-CDs, XRD analysis was performed with a D8-Advance instrument from Bruker (Karlsruhe, Germany). Moreover, the morphology, size, and microstructure of the carbon dots were examined using a transmission electron microscope (F200; JEOL, Tokyo, Japan).

### 4.5. In Vitro Biocompatibility of SC-CDs

#### 4.5.1. Hemocompatibility of SC-CDs

A hemolysis assay was used to evaluate the hemocompatibility of the SC-CDs. In brief, 5 mL of anticoagulated whole blood was collected from a male SD rat weighing 200 g and subjected to centrifugation at 3000 rpm in a high-speed refrigerated centrifuge for 10 min to separate the upper layer of the plasma. The cells were washed twice with physiological saline until a clear and transparent supernatant was obtained, which was discarded to isolate the packed red blood cells. The cells were diluted with phosphate-buffered saline at a ratio of 1:1 to yield a red blood cell suspension. Subsequently, 1.6, 2.0, and 4.0 mg of the SC-CDs were added to 200 μL of the red blood cell suspension, thoroughly mixed, and incubated for 1 h. After this step, 5 mL of physiological saline was added to each group, and the solutions were mixed using a vortex and subsequently centrifuged at 3000 r/min for 10 min. The resulting supernatant (200 μL) was collected, and the absorbance at 545 nm was measured using a microplate reader (BIO-TEK ELX800, Molecular Devices Shanghai Co., Ltd., Shanghai, China). Deionized water served as the positive control, whereas blank wells acted as the negative control.

Hemolysis rate (%) = [(OD_sam_ − OD_con_)/(OD_dw_ − OD_0_)] × 100,

where OD_sam_, OD_con_, OD_dw,_ and OD_0_ represent the absorbance of the sample, negative control, positive control, and background in the 96-well plate, respectively.

#### 4.5.2. Cytotoxicity of SC-CDs in L929 Cells

The cytotoxicity of SC-CDs was assessed in L929 fibroblast cells maintained in DMEM containing 10% fetal bovine serum. The cells were maintained at 37 °C in a 5% CO_2_ environment. Upon reaching the third passage, the L929 cells were seeded into 96-well plates at a density of 10,000 cells per well and incubated for 24 h to allow for attachment. SC-CDs solutions at concentrations of 4000, 2000, 1000, 500, 250, 125, and 63 μg/mL were prepared using serum-free DMEM. These solutions were added to the wells to achieve final concentrations of 2000, 1000, 500, 250, 125, 63, and 31 μg/mL, respectively.

Cell viability was assessed after 24, 48, and 72 h by replacing the medium with 100 μL of a 1 mg/mL 3-(4,5-dimethylthiazol-2-yl)-2,5-diphenyltetrazolium bromide (MTT) solution prepared in DMEM, followed by incubation for 4 h in a cell culture incubator. After removing the MTT solution, the absorbance at 570 nm was measured using 100 μL dimethyl sulfoxide (DMSO).

Cell viability (%) = (A_sam_/A_con_) × 100

A_sam_ and A_con_ represent the absorbance at 570 nm for the experimental and control groups, respectively.

### 4.6. In Vitro Bioactivity

#### 4.6.1. In Vitro Hemostatic Activity

Coagulation parameters, including the APTT, PT, TT, and FIB levels, were analyzed using a fully automated coagulation analyzer (RAC-30; Shenzhen Ledu Life Science Co., Ltd., Shenzhen, Guangdong, China). An LDH assay kit was used to detect the platelet aggregation-promoting effect of the SC-CDs. Platelet-rich plasma was diluted to a concentration of 5 × 10^8^/mL and the SC-CDs were added to obtain final concentrations of 1, 2, and 5 mg/mL. The mixture was incubated at 37 °C for 30 min and washed three times with phosphate-buffered saline (PBS) to remove the unattached platelets. The mixture was treated with 200 μL of 2% Triton X-100 and incubated at 37 °C for 1 h to induce platelet lysis. Subsequently, the LDH supernatant was collected via centrifugation at 10,000 r/min for 15 min, and the LDH activity was measured using the LDH assay kit to determine the OD_440_ value of each sample.

LDH activity (U/L) = [(OD_measured_ − OD_control_)/(OD_standard_ − OD_blank_)] × C_standard_ × 100

OD_measured_ represents the absorbance value of the sample being tested, OD_control_ represents the absorbance of the control, OD_standard_ represents the absorbance value for Triton X-100, and OD_blank_ represents the absorbance value of the blank reagent.

Platelet activation was assessed using a flow cytometry analyzer (Accuri^TM^ C6 Plus, Becton, Dickinson and Company, San Jose, CA, USA). A total of 10^7^ platelets were incubated with the SC-CDs (1, 2, and 5 mg/mL) at 37 °C for 30 min. After centrifugation at 1200× *g* for 10 min, the supernatant was discarded, and the pellet was resuspended in 1 mL of PBS and gently mixed through pipetting. PE-CD61 (1.25 μL) and APC-CD62P (2.5 μL) were added to the mixture, which was then incubated in the dark at 4 °C for 20 min. Quantification of activated platelets was performed using flow cytometry.

#### 4.6.2. Cell Scratch Assay

L929 cells (400,000) were seeded into a standard-sized six-well plate for an initial period of 24 h. To create reproducible scratches on the monolayer cell surface, we introduced a precise scratch using a 10 μL pipette tip. The cells were incubated with the SC-CDs at an optimized concentration of 500 μg/mL at 37 °C. Images were captured periodically at three distinct time points (24 h, 48 h, and 72 h) to visually assess and quantify the degree of scratch closure. All images were analyzed using specialized ImageJ2 software.

### 4.7. In Vivo Hemostatic Activity

#### 4.7.1. Rat Liver Injury Bleeding Model

Nine male SD rats, each weighing approximately 200 g, were randomly allocated into three distinct groups: Control, SC-CDs, and YNBY groups. Following anesthesia, the abdominal fur was shaved and a section of the peritoneum was incised to expose the left lower lobe of the liver. A sheet of filter paper was positioned beneath the liver lobe, and a needle was used to create a 1 cm long and 0.3 cm deep puncture wound on the liver lobe. After allowing free bleeding for 5 s, 30 mg of YNBY or SC-CDs powder was sprinkled onto the wound. Subsequently, a sterile compress was placed over the wound, and constant pressure with a weight of 50 g was applied to achieve hemostasis in the liver lobe. The time required for hemostasis as well as the amount of bleeding were recorded for each experiment, which was repeated three times.

Bleeding volume (g) = M_1_ − M_0_,

where M_1_ represents the weight of the filter paper and gauze after the cessation of blood flow, and M_0_ refers to the initial weight of the filter paper and gauze.

#### 4.7.2. Rat Tail Amputation Bleeding Model

Nine male SD rats, each weighing 200 g, were randomly assigned to three groups: Control, SC-CDs, and YNBY groups, for the tail bleeding experiment. After amputating the tail at its midpoint, 20 mg of SC-CDs or YNBY powder was immediately sprinkled onto the incision site. The tail was allowed to bleed naturally, and blood samples were collected using an EP tube. The time to bleeding cessation was recorded, and the experiment was repeated three times for each group.

#### 4.7.3. Mouse Snake Venom Bleeding Model

The inhibitory effect of the carbon dots on internal bleeding was assessed using a snake venom-induced bleeding model. Male BALB/c mice weighing approximately 20 g were divided into six groups: Control, SV, hemocoagulase (HC), low-dose SC-CDs (L-CD, 2 mg/kg), medium-dose SC-CDs (M-CD, 4 mg/kg), and high-dose SC-CDs (H-CD, 8 mg/kg) groups, with each group comprising three mice. Various concentrations of the SC-CDs were intraperitoneally administered to the mice, followed by an intraperitoneal injection of snake venom (2 mg/kg). The Control group served as the negative control and only received normal saline, whereas the SV group served as the positive control and received snake venom along with an equal volume of normal saline. The HC group was treated with snake venom and received an i.p. of HC at a dose of 1 kU/kg. One hour after administration, the mice were euthanized to assess the extent of bleeding in the peritoneal cavity. Subsequently, 3 mL of ultrapure water was added to wash and quantify the bleeding from the abdominal organs.

### 4.8. In Vivo Wound-Healing Effect of SC-CDs

#### 4.8.1. Complete Cortical Defect Model Construction

Sixty male BALB/c mice weighing 18–20 g were randomly divided into five groups: a negative control (Control) group treated with normal saline, and L-CD (0.25 mg/kg), M-CD (0.5 mg/kg), H-CD (1 mg/kg), and positive control (YNBY) groups, with 12 mice in each group. The mice were anesthetized using an intraperitoneal injection of pentobarbital sodium (1%, 0.1 mL/20 g), and a complete full-thickness skin defect measuring 10 × 10 mm^2^ was marked and created on the back of each mouse after shaving and disinfecting with a 75% ethanol and iodine solution. Each wound in every group was topically administered with either normal saline, the SC-CDs (0.25, 0.5, and 1 mg/mL), or a 1 mg/mL YNBY solution. Subsequently, sterile gauze was applied to the wounds, which were then moistened every 4 h using their respective solutions until natural healing occurred on the seventh day.

#### 4.8.2. Wound Area Change in the Mouse Model

The wound area for each group was captured and documented on days 3, 7, and 14. Precise measurements of the wound area were performed using Image J2 software, whereas the wound healing rate was calculated using the following formula:

Wound area (%) = S_1_/S_0_ × 100

S_1_ represents the wound area at the time of measurement, whereas S_0_ corresponds to the initial area.

#### 4.8.3. HE Staining and Histopathological Scoring of the Wound Surface

The animals were euthanized on days 0 and 14 for histopathological examination. Tissue specimens were obtained by excising the skin, which was then fixed in 4% paraformaldehyde for subsequent HE staining, Masson staining, and immunohistochemical analysis.

### 4.9. Statistical Analysis

The data were analyzed using IBM SPSS Statistics 26 (SPSS Inc., Chicago, IL, USA) and GraphPad Prism 8.0 (GraphPad Software Inc., San Diego, CA, USA). The results are presented as the mean ± standard deviation. Differences between groups were assessed for statistical significance using two-way analysis of variance or an unpaired *t-*test. A *p*-value of <0.05 was considered statistically significant.

## 5. Conclusions

In this study, we synthesized SC-CDs through a hydrothermal method and examined their efficacy in hemostasis and wound healing. The SC-CDs featured a negatively charged surface that plays an important role in promoting hemostasis. This surface can activate platelets, thereby enhancing their adhesion and aggregation, which subsequently facilitates blood clot formation. Furthermore, it stimulates the endogenous coagulation pathway, expediting the clotting process resulting from internal bleeding, traumatic injuries, and coagulation disorders. In addition to their hemostatic properties, SC-CDs exhibit antioxidant capabilities and promote fibroblast proliferation and migration, thereby accelerating the wound-healing process. These attributes suggest that SC-CDs have potential as effective injectable nanomedicines for managing traumatic bleeding, coagulation disorders, and tissue injuries. However, before considering their clinical application, it is imperative to thoroughly elucidate the molecular mechanisms underlying their effect on clotting processes and address any long-term safety concerns. Overall, the environmentally friendly synthesis of biocompatible SC-CDs enhances the material foundation for carbon-based hemostasis while offering a novel therapeutic approach for addressing bleeding episodes and tissue trauma.

## Figures and Tables

**Table 1:** Synthesis methods and applications of various carbon dots derived from natural sources.

Number	Precursor	Synthesis	Application	Ref.
1	*Platycladus orientalis*	Hydrothermal methods	Hemostasis	[33]
2	*Ligusticum wallichii*		Hemostasis, wound healing	[34]
3	Berberine		Anticancer	[35]
4	Ascorbic acid		Antibacterial	[36]
5	*Osmanthus fragrans* lours		Biosensing	[37]
6	Degummed silk		Antibacterial, antioxidant	[38]
7	Silkworm cocoon	High-temperature pyrolysis methods	Anti-inflammatory	[39]
8	Pollen typhae		Anti-inflammatory, antioxidant	[40]
9			Hemostasis	[41]
10	Lychee seeds		Bioimaging	[42]
11	Poly-L-lysine and oxidized dextran		Antibacterial, wound healing	[18]
12	Carboxymethyl chitosan	Microwave methods	Antioxidant	[43]
13	*Elaeagnus angustifolia*		Antibacterial	[44]
14	Chitosan and *o*-phenylenediamine		Bioimaging	[45]

## Data Availability

Data are contained within the article.

## Supplementary Materials

The following supporting information can be downloaded at: https://www.mdpi.com/article/10.3390/ph18050603/s1. ARRIVE compliance questionnaire. Figure S1. Quantum total yield (A) and hemostasis time (B) of SC-CDs prepared without temperature control, and the quantum total yield (C) and hemostasis time (D) of SC-CDs prepared at 180 °C. Figure S2. Raman spectra (A) and zeta potential (B) of SC-CDs. Figure S3. Scavenging rate of SC-CDs for ABTS (A) and O_2_^−^ (B).

## Abbreviations

The following abbreviations are used in this manuscript:

SC-CDs	silkworm cocoon-derived carbon dots
APTT	activated partial thromboplastin time
PT	prothrombin time
TT	thrombin time
FIB	fibrinogen
UV	ultraviolet
FTIR	Fourier-transform infrared spectroscopy
XPS	X-ray photoelectron spectroscopy
XRD	X-ray diffraction
LDH	lactate dehydrogenase
YNBY	Yunnan Baiyao
SV	snake venom
HC	hemocoagulase
HR-TEM	high-resolution transmission electron microscopy
QY	quantum yield
ROS	reactive oxygen species
DMEM	Dulbecco’s Modified Eagle Medium
MTT	3-(4,5-dimethylthiazol-2-yl)-2,5-diphenyltetrazolium bromide
DMSO	dimethyl sulfoxide
