# Supplementary material for: Silkworm Cocoon—Derived Carbon Dots for Post-Trauma Hemostasis and Tissue Repair
Source: Pharmaceuticals (Basel). 2025 Apr 22;18(5):603. doi: 10.3390/ph18050603 (PMC12114995; doi:10.3390/ph18050603)
Supplement: Supplementary file 1 [file pharmaceuticals-18-00603-s001.zip › pharmaceuticals-3551452-supplementary figures.pdf]

Supplementary information

Silkworm Cocoon-Derived Carbon Dots for Post-Trauma Hemostasis and Tissue Repair

Xinru Wu [1], Miaomiao Yao [1,2], Xuan Qiao [1], Lintao Li [1], Zhiyun Meng [1], Shuchen Liu [1], Yunbo Sun [1], Hui Gan [1], Xiaoxia Zhu [1], Zhuona Wu [1], Ruolan Gu [1,*], Guifang Dou [1,*]

[1] Beijing Institute of Radiation Medicine, Beijing 100850, China
[2] School of Pharmaceutical Sciences, Anhui Medical University, Hefei 230032, China
* Correspondence: guruolan@bmi.ac.cn (R.G.); dougf@bmi.ac.cn (G.D.)

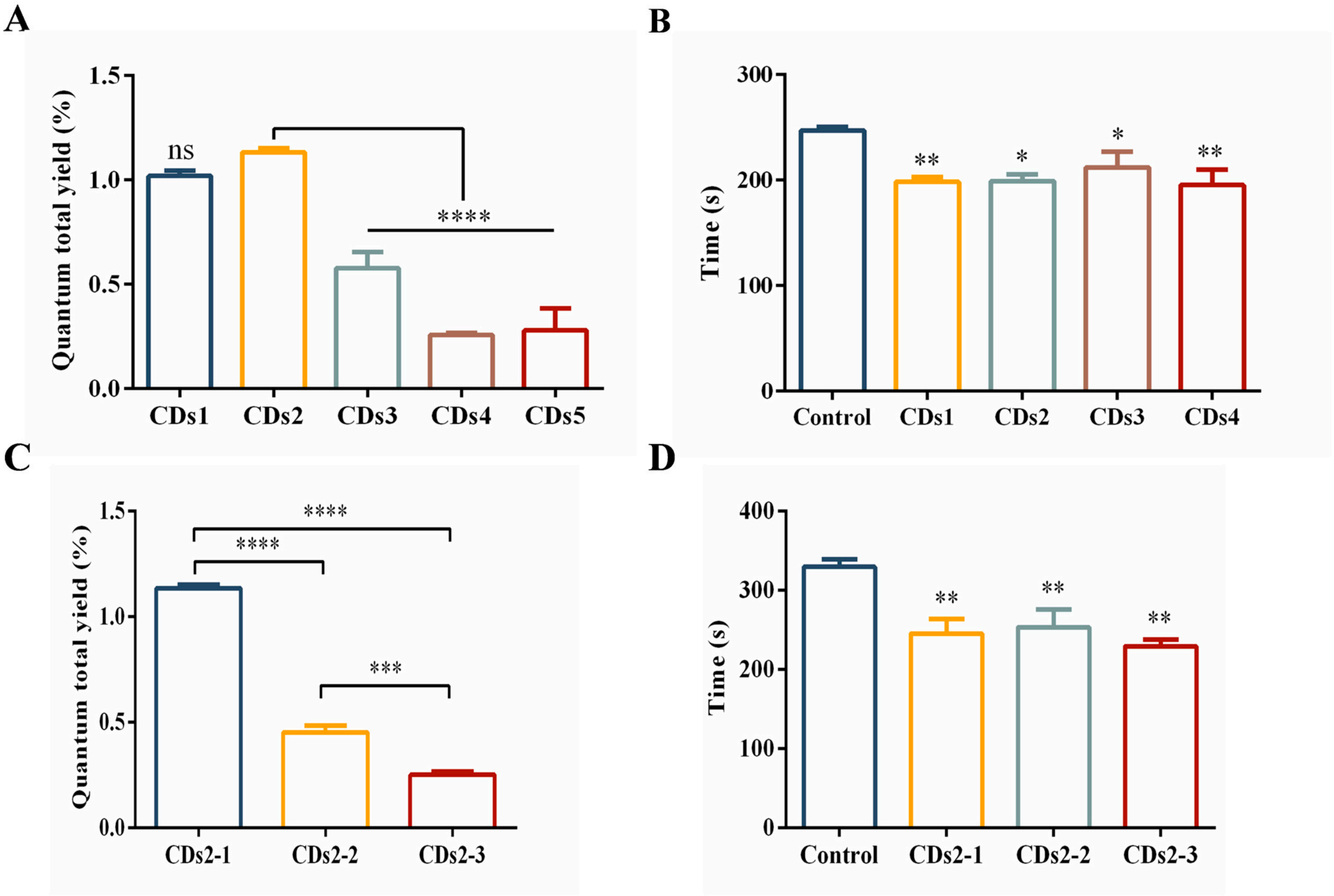

Figure S1. Quantum total yield (A) and hemostasis time (B) of SC-CDs prepared without temperature control, and the quantum total yield (C) and hemostasis time (D) of SC-CDs prepared at different temperatures of 180°C.

(CDs1, CDs2, CDs3, and CDs4 represent samples at 160°C, 180°C, 200°C, and 220°C, respectively.

CDs2-1, CDs2-2, and CDs2-3 represent samples prepared at a temperature of 180°C for 6 hours, 8 hours, and 10 hours, respectively.

*p <0.05, **p <0.01, ***p <0.001, ****p <0.0001)

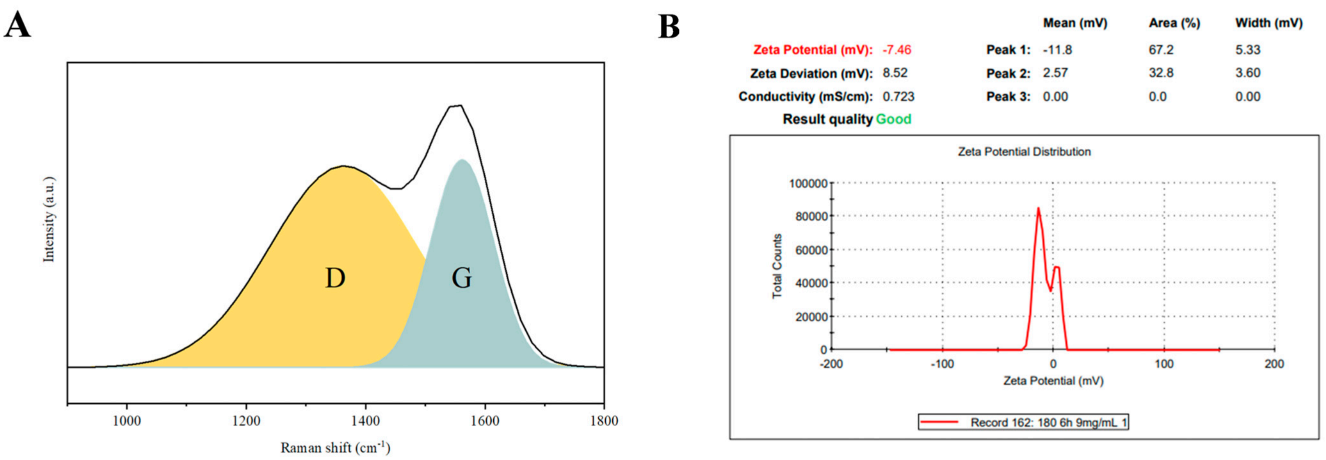

Figure S2. Raman spectra (A) and zeta potential (B) of SC-CDs

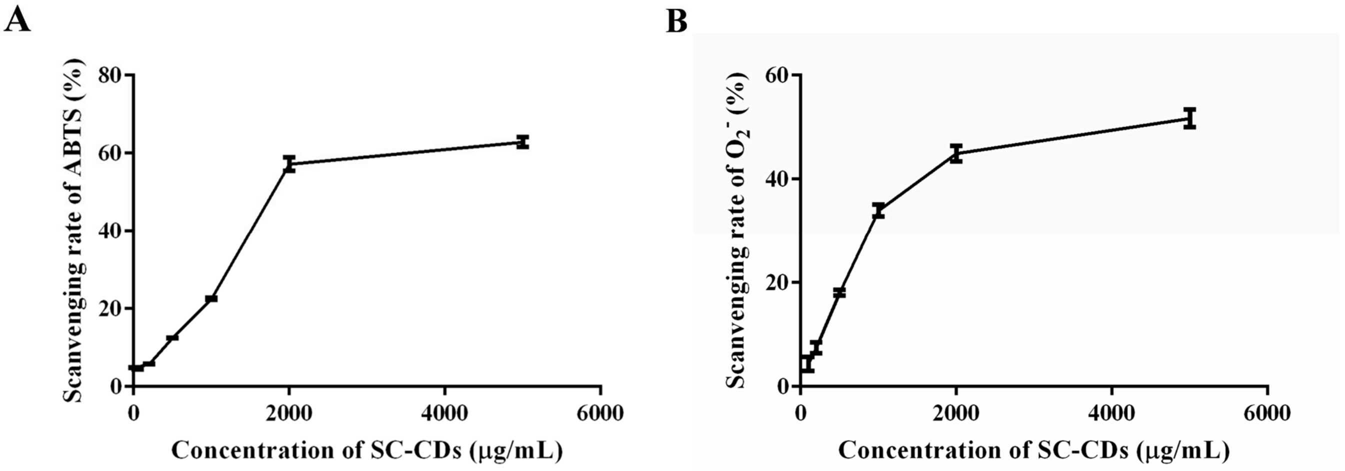

Figure S3. Scavenging rate of SC-CDs for ABTS (A) and O_2^- (B)
